# Molecular and clinical analyses of HTLV-1 subgroups associated with the risk of developing HAM/TSP.

**DOI:** 10.1186/1742-4690-12-S1-P23

**Published:** 2015-08-28

**Authors:** Mineki Saito, Keiko Yasuma, Tadasuke Naito, Toshio Matsuzaki, Yuichi Mitobe, Yoshihisa Yamano, Hiroshi Takashima, Masao Matsuoka

**Affiliations:** 1 Department of Microbiology, Kawasaki Medical School, Okayama, Japan; 2 Laboratory of Virus Control, Institute for Virus Research, Kyoto University, Kyoto, Japan; 3 Department of Neurology and Geriatrics, Kagoshima University Graduate School of Medical and Dental Sciences, Kagoshima, Japan; 4 Department of Rare Diseases Research, Institute of Medical Science, St. Marianna University School of Medicine, Kanagawa, Japan

## 

Among human T-lymphotropic virus type 1 (HTLV-1)-infected individuals, the lifetime risk of developing HTLV-1-associated myelopathy/tropical spastic paraparesis (HAM/TSP) differs between ethnic groups, also, there is an association between HTLV-1 tax gene subgroups (i.e., tax-A or -B) and the risk of HAM/TSP. In an attempt to understand the molecular mechanism responsible for this association, we studied the full-length proviral genome sequences of various HTLV-1-infected cell lines and patient samples, the functional difference in the viral transcriptional regulator Tax and HBZ between each subgroup, and the relationship between tax subgroups and the clinical and laboratory characteristics of HAM/TSP patients. The results indicated that (1) the distinct nucleotide substitutions corresponding to each tax subgroup are also associated with the nucleotide substitutions of viral structural, regulatory, and accessory genes; (2) mRNA expression of HBZ in HTLV-1-infected cells was significantly higher in HAM/TSP patients with tax-B than those with tax-A; (3) there was a positive correlation between HBZ and the expression of its target FoxP3 in HAM/TSP patients with tax-B but not in patients with tax-A; (4) transcriptional changes in inducible cells that express each subgroup of Tax or HBZ protein under the control of an inducible promoter showed different target gene profiles; however, (5) there were no clear differences in clinical and laboratory characteristics between HAM/TSP patients with tax-A and tax-B; and (6) there were no functional differences in Tax and HBZ between each subgroup evaluated by reporter gene assays. Our results indicate that although different HTLV-1 tax subgroups cause different patterns of viral and host gene expression in HAM/TSP patients via independent mechanisms of direct transcriptional regulation, these differences do not significantly affect the clinical and laboratory characteristics of HAM/TSP patients. Further studies on other viral genes, as well as clinical samples of asymptomatic carriers and adult T-cell leukemia (ATL) patients, are in progress to clarify the role of HTLV-1 tax subgroups.

